# High-quality genome sequence of the radioresistant bacterium *Deinococcus ficus* KS 0460

**DOI:** 10.1186/s40793-017-0258-y

**Published:** 2017-07-28

**Authors:** Vera Y. Matrosova, Elena K. Gaidamakova, Kira S. Makarova, Olga Grichenko, Polina Klimenkova, Robert P. Volpe, Rok Tkavc, Gözen Ertem, Isabel H. Conze, Evelyne Brambilla, Marcel Huntemann, Alicia Clum, Manoj Pillay, Krishnaveni Palaniappan, Neha Varghese, Natalia Mikhailova, Dimitrios Stamatis, TBK Reddy, Chris Daum, Nicole Shapiro, Natalia Ivanova, Nikos Kyrpides, Tanja Woyke, Hajnalka Daligault, Karen Davenport, Tracy Erkkila, Lynne A. Goodwin, Wei Gu, Christine Munk, Hazuki Teshima, Yan Xu, Patrick Chain, Michael Woolbert, Nina Gunde-Cimerman, Yuri I. Wolf, Tine Grebenc, Cene Gostinčar, Michael J. Daly

**Affiliations:** 1 Uniformed Services University of the Health Sciences, School of Medicine, Bethesda, MD USA; 2 Henry M. Jackson Foundation for the Advancement of Military Medicine, Bethesda, MD USA; 3 National Center for Biotechnology Information, National Library of Medicine, National Institutes of Health, Bethesda, MD USA; 4 University of Bielefeld, Bielefeld, Germany; 5 Leibniz Institute DSMZ - German Collection of Microorganisms and Cell Cultures, Braunschweig, Germany; 6 DOE Joint Genome Institute, Walnut Creek, CA USA; 7 Los Alamos National Laboratory, Los Alamos, NM USA; 8 Department of Biology, Biotechnical Faculty, University of Ljubljana, Ljubljana, Slovenia; 9 Slovenian Forestry Institute, Ljubljana, Slovenia

**Keywords:** *Deinococcus-Thermus*, *Deinococcaceae*, *Deinococcus ficus*, Radiation-resistant, Rod-shaped, Phenotype characterization, Genome analysis, Phylogenetic analysis

## Abstract

The genetic platforms of *Deinococcus* species remain the only systems in which massive ionizing radiation (IR)-induced genome damage can be investigated in vivo at exposures commensurate with cellular survival. We report the whole genome sequence of the extremely IR-resistant rod-shaped bacterium *Deinococcus ficus* KS 0460 and its phenotypic characterization. *Deinococcus ficus* KS 0460 has been studied since 1987, first under the name *Deinobacter grandis*, then *Deinococcus grandis*. The *D. ficus* KS 0460 genome consists of a 4.019 Mbp sequence (69.7% GC content and 3894 predicted genes) divided into six genome partitions, five of which are confirmed to be circular. Circularity was determined manually by mate pair linkage. Approximately 76% of the predicted proteins contained identifiable Pfam domains and 72% were assigned to COGs. Of all *D. ficus* KS 0460 proteins, 79% and 70% had homologues in *Deinococcus radiodurans* ATCC BAA-816 and *Deinococcus geothermalis* DSM 11300, respectively. The most striking differences between *D. ficus* KS 0460 and *D. radiodurans* BAA-816 identified by the comparison of the KEGG pathways were as follows: (i) *D. ficus* lacks nine enzymes of purine degradation present in *D. radiodurans*, and (ii) *D. ficus* contains eight enzymes involved in nitrogen metabolism, including nitrate and nitrite reductases, that *D. radiodurans* lacks. Moreover, genes previously considered to be important to IR resistance are missing in *D. ficus* KS 0460, namely, for the Mn-transporter *nramp*, and proteins DdrF, DdrJ and DdrK, all of which are also missing in *Deinococcus deserti*. Otherwise, *D. ficus* KS 0460 exemplifies the *Deinococcus* lineage.

**Electronic supplementary material:**

The online version of this article (doi:10.1186/s40793-017-0258-y) contains supplementary material, which is available to authorized users.

## Introduction

Species of the genus *Deinococcus* have been studied for their extreme IR resistance since the isolation of *Deinococcus radiodurans* in 1956 [1]. Since then, many other species of the same genus have been isolated. The current number of recognized *Deinococcus* species is greater than 50 while there are more than 300 non-redundant 16S rRNA sequences of the family *Deinococcaceae* in the ARB project database [2]. Apart from *Deinococcus ficus* KS 0460, only a few other representatives have been studied in detail for their oxidative-stress resistance mechanisms: *D. radiodurans*, *Deinococcus geothermalis* and *Deinococcus deserti* [3]*.* The picture that has emerged for the life cycle of most *Deinococcus* species is one comprised of a cell-replication phase that requires nutrient-rich conditions, such as in the gut of an animal, followed by release, drying and dispersal [1]. Desiccated deinococci can endure for years, and, if blown by winds through the atmosphere, are expected to survive and land worldwide. As reported, some deinococci become encased in ice, and some entombed in dry desert soils. High temperatures also are not an obstacle to the survival of some deinococcal species. *D. geothermalis* and *Deinococcus murrayi* were originally isolated from hot springs in Italy and Portugal, respectively [1]. The prospects of harnessing the protective systems of *D. radiodurans* for practical purposes are now being realized.

The complete genome sequence presented here is for *D. ficus* KS 0460, originally named *Deinobacter grandis* KS 0460, isolated in 1987 from feces of an Asian elephant (*Elephas maximus*) raised in the Ueno Zoological Garden, Tokyo, Japan (Table 1) [4]. Later, *Deinobacter grandis* was renamed *Deinococcus grandis* [5]. Strain KS 0460 was acquired by USUHS from the originating laboratory in 1988 by Kenneth W. Minton and has been the subject of study here ever since. As a candidate for bioremediation of radioactive DOE waste sites [6] and a target of study for DNA repair [7], *D. ficus* KS 0460 was chosen for whole genome sequencing. The *D. ficus* KS 0460 genome now adds to the growing number of sequenced *Deinococcus* species needed to decipher the complex extreme IR resistance phenotype. To date, a genetic explanation for the complex survival tactics of deinococci has not been provided by comparative genomics or transcriptomics [8].

**Table 1:** Classification and general features of *Deinococcus ficus* KS 0460 according to MIGS recommendations [49]

MIGS ID	Property	Term	Evidence code^a^
	Classification	Domain *Bacteria*	TAS [50]
		Phylum *Deinococcus-Thermus*	TAS [51, 52]
		Class *Deinococci*	TAS [53, 54]
		Order *Deinococcales*	TAS [5]
		Family *Deinococcaceae*	TAS [5, 55]
		Genus *Deinococcus*	TAS [5, 55]
		Species *Deinococcus ficus*	TAS [4, 9]
		Strain: KS 0460	
	Gram stain	Variable	TAS [4, 9]
	Cell shape	Rod	TAS [4, 9]
	Motility	Non-motile	TAS [4, 9]
	Sporulation	None	TAS [4, 9]
	Temperature range	Mesophile	TAS [4, 9]
	Optimum temperature	30-37 °C	TAS [4, 9]
	pH range; Optimum	e.g. 5.5–10.0; 7.0	TAS [4, 9]
	Carbon source	Glucose, fructose	TAS [9]
MIGS-6	Habitat	*Elephas maximus* feces	TAS [4]
MIGS-6.3	Salinity	1% NaCl (*w*/*v*)	TAS [4]
MIGS-22	Oxygen requirement	Aerobic	TAS [4]
MIGS-15	Biotic relationship	Free-living	NAS
MIGS-14	Pathogenicity	Non-pathogen	NAS
MIGS-4	Geographic location	Tokyo/Japan	TAS [4]
MIGS-5	Sample collection	1987	TAS [4]
MIGS-4.1	Latitude	Non reported	
MIGS-4.2	Longitude	Non reported	
MIGS-4.4	Altitude	Non reported	

^a^Evidence codes - IDA: Inferred from Direct Assay; TAS: Traceable Author Statement (i.e., a direct report exists in the literature); NAS: Non-traceable Author Statement (i.e., not directly observed for the living, isolated sample, but based on a generally accepted property for the species, or anecdotal evidence). These evidence codes are from the Gene Ontology project [56]

## Organism information

### Classification and features

**Fig. 1:**
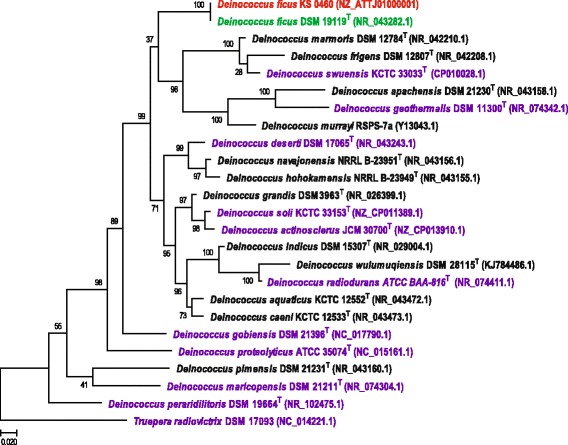
16S rRNA phylogenetic tree of the *Deinococcus* genus. The multiple alignment of 16S rRNA sequences was constructed using MUSCLE program [58] with default parameters. The maximum-likelihood phylogenetic tree was reconstructed using the FastTree program [59], with GTR substitution matrix and gamma-distributed evolutionary rates. The same program was used to compute bootstrap values. *Truepera radiovictrix* was chosen as an outgroup. *D. ficus* KS 0460 is marked in *red*, *D. ficus* DSM 19119/CC-FR2-10 [9] - in *green*, completely sequenced according to NCBI genomes - in *purple*

In a chemotaxonomic study published in 1987, an isolate (strain KS 0460) from γ-irradiated feces of an Asian elephant yielded an IR-resistant bacterium with a wall structure, cellular fatty acid composition, and GC content typical of members of the genus *Deinococcus* [4]. However, strain KS 0460 was rod-shaped and grew as pink-pigmented colonies, whereas most other deinococci grow as diplococci/tetracocci and yield red colonies. The original isolate was named *Deinobacter grandis*, but was later renamed *Deinococcus grandis* based on its close phylogenetic relationship (16S rRNA sequences) with deinococci [5]. Strain KS 0460 was subsequently included in experimental IR survival studies together with other *Deinococcus* species, where it was referred to as *grandis* [7]. Our 16S rRNA phylogenetic analysis confirms that strain KS 0460 belongs to the genus *Deinococcus*, most closely related to the type strain of *Deinococcus ficus* DSM 19119 (also referred to as CC-FR2-10) (Fig. 1).

**Fig. 2:**
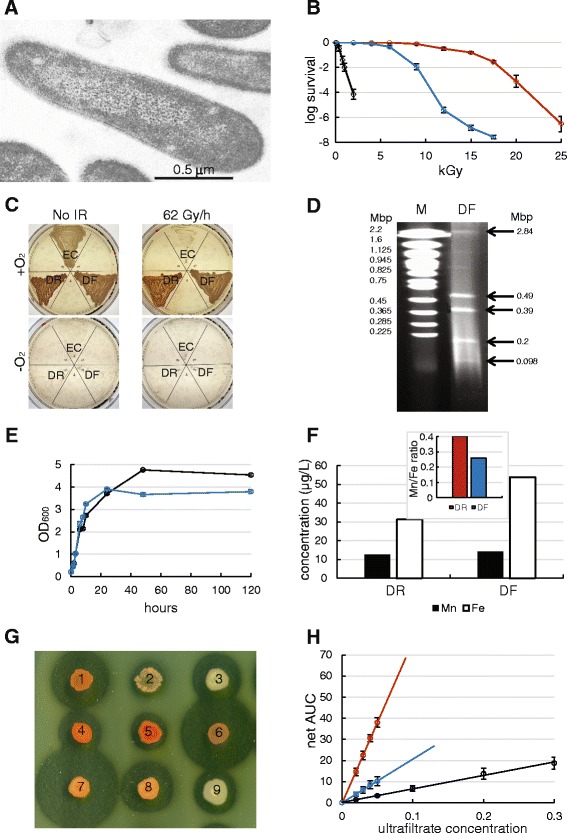
*Deinococcus ficus* KS 0460 (EXB L-1957) phenotype. **a** Transmission electron micrograph. *D. ficus* grown in TGY, early-stationary phase. **b** Survival of *D. radiodurans* BAA-816 (red), *D. ficus* (*blue*), and *E. coli* (strain K-12, MG1655) (*black*) exposed to acute IR. The indicated strains were inoculated in liquid TGY and grown to OD_600_ ~ 0.9. Cells were then irradiated on ice with Co-60. **c** *D. ficus* is an aerobe capable of growth under 62 Gy/h. DR, *D. radiodurans*; DF, *D. ficus*; EC, *E. coli*. **d** PFGE of genome partitions in a 0.9% agarose gel. PFGE conditions: 0.5 × TBE, 6 V/cm with a 10 to 100 s switch time ramp at an included angle of 120°, 14 °C, 18 h. M, marker *S. cerevisiae* YNN (BioRad). **e** Growth curves at 37 °C (*blue*) and 32 °C (*black*) in TGY medium. **f** ICP-MS on Mn and Fe content of *D. radiodurans* BAA-816 and *D. ficus*. Inset: Mn/Fe ratios. **g** Protease secretion assay. Halos indicate activity of proteases [60]. Strains: 1. *D. radiodurans* BAA-816, 2. *D. geothermalis* DSM 11300, 3. *D. ficus* KS 0460, 4. *D. murrayi* (MD591), 5. *D. radiopugnans* (MD567), 6. *D. radiodurans* (MD878, SX-108-7B-1, [61]), 7. *D. proteolyticus* (MD568), 8. *D. proteolyticus* (MD628, [62]), and 9. *D. proteolyticus* (MD869). **h** Antioxidant capacities of *D. radiodurans* BAA-816 (*red*), *D. ficus* (*blue*), and *E. coli* (strain K-12, MG1655) (*black*) ultrafiltrates assessed by antioxidant assay as described previously [63, 64]. Net AUC is an integrative value of a total fluorescence during antioxidant reaction in the presence of ultrafiltrates

Consistent with the original description of *D. ficus* KS 0460, the rod-shaped cells are 0.5 to 1.2 μm by 1.5 to 4.0 μm (Fig. 2a) and grow as pink colonies [4, 9]. *D. ficus* KS 0460 was shown to have a D_10_ of approximately 7 kGy (Co-60) (Fig. 2b) and is capable of growth under chronic γ-irradiation at 62 Gy/h (Cs-137) (Fig. 2c). The cells are aerobic, incapable of growth under anaerobic conditions on rich medium, irrespective of the presence or absence of chronic IR (Fig. 2c). The general structure of the *D. ficus* KS 0460 genome was analyzed by PFGE of genomic DNA prepared from embedded cells. The plugs containing digested cells were exposed to 200 Gy prior to electrophoresis, a dose gauged in vitro to induce approximately 1 DNA double strand break per chromosome in the range 0.5 - 2 Mbp [10]. Fig. 2d shows the presence of the five largest genomic partitions: main chromosome (~2.8 Mbp), 3 megaplasmids (~500 kb, ~400 kb and ~200 kbp) and one plasmid (~98 kbp), predicting a genome size ~4.0 Mbp. We did not observe the smallest genome partition (0.007 Mbp) by PFGE. The growth characteristics of *D. ficus* KS 0460 in liquid culture at 32 and 37 °C (Fig. 2e) are very similar to *D. radiodurans* [11]. It is unknown if strain *D. ficus* KS 0460 is genetically tractable because the cells are naturally resistant to the antibiotics tetracycline, chloramphenicol and kanamycin at concentrations needed to select for plasmids and integration vectors designed for *D. radiodurans* [12] (data not shown). *D. ficus* KS 0460, like other deinococci, accumulate high concentrations of Mn^2+^ (Fig. 2f) [7, 13]. Bacterial Mn^2+^ accumulation was previously shown to be important to extreme IR resistance, mediated by the Mn transport gene *nramp* and ABC-type Mn-transporter gene [14]. We also showed that *D. ficus* KS 0460 produces proteases, as detected in a protease secretion assay on an indicator plate containing skimmed milk (Fig. 2g). For example, in *D. radiodurans*, the products of proteases – peptides – form Mn^2+^-binding ligands of *Deinococcus* Mn antioxidants, which protect proteins from IR-induced ROS, superoxide in particular [8, 13, 15]. Finally, we show that *D. ficus* KS 0460 cells have a high intracellular antioxidant capacity (Fig. 2h), which is a strong molecular correlate for IR resistance [1, 11].

#### Extended feature descriptions

16S rDNA gene phylogenetic analysis was based on sequences from 22 type strains of genus *Deinococcus* including ten from completely sequenced genomes, and two from *Deinococcus ficus* strains KS 0460 and DSM 19119; and *Truepera radiovictrix* DSM 17093, the distinct species shown to be an outgroup to the *Deinococcus* genus [16]. The maximum-likelihood phylogenetic trees were reconstructed using two approaches: (i) the FastTree program [17], with GTR substitution matrix and gamma-distributed evolutionary rates and maximum-likelihood algorithm; and (ii) PHYML program with the same parameters (Fig. 1 and Additional file 1: Figure S1) [18]. Both *D. ficus* strains, as expected, group together, but the position of this pair in both trees is poorly resolved (37 support value for FastTree method and 44 for PHYML method) potentially because of the long branch of this clade. In both trees, however, the *D. ficus* clade confidently groups deep in the *Deinococcus* tree within the branch with *D. gobiensis* as a sister clade.

## Genome sequencing information

### Genome project history

*Deinococcus ficus* KS 0460 was obtained from the Oyaizu laboratory and was entered into the Daly strain collection at USUHS on November 18, 1997. The strain was submitted to the EX Culture Collection, Mycosmo, Slovenia, on December 29, 2016 and was issued an accession number EXB L-1957. The genome of *D. ficus* KS 0460 was sequenced at the JGI. The project was initiated in 2009, the genome was released on August 26, 2012 as “*Deinococcus* sp. 2009”. The genome of *D. ficus* KS 0460 has the status of an improved high-quality draft. The genome assembly and annotation can be accessed through the JGI genome portal [19] and also GenBank [20]. The genome is considered to be near-complete. The search for bacterial Benchmarking Universal Single-Copy Orthologs [21] found a comparable number of orthologs in *D. ficus* KS 0460 and in ten complete *Deinococcus* species genomes. Furthermore, of the 875 genes representing the core genome of the same ten complete *Deinococcus* species as determined by the GET_HOMOLOGUES pipeline [22], only five genes were missing from *D. ficus* KS 0460.

### Growth conditions and genomic DNA preparation

*D. ficus* KS 0460 was recovered from a glycerol frozen stock on TGY solid rich medium (1% bactotryptone, 0.1% glucose, and 0.5% yeast extract, 1.5% *w*/*v* bacto agar) (3 days, 32 °C) with following inoculation of 25 ml TGY medium. The culture was grown up to OD_600_ ~ 0.9. Subsequently, 19 ml were used to inoculate 2 L of TGY medium and the culture was grown at 32 °C, overnight in aerated conditions in a shaker incubator (200 rpm). The cells were harvested at OD_600_ ~ 1.6. The DNA was isolated from a cell pellet (5.6 g) using Jetflex Genomic DNA Purification Kit (GENOMED, Germany). The final DNA concentration was 80 μg ml^−1^, in a volume of 800 μl. The DNA was RNA free and passed quality control.

### Genome sequencing and assembly

The draft genome of *D. ficus* KS 0460 was generated at the JGI using Illumina data (Table 2) [23]. Two paired-end Illumina libraries were constructed, one short-insert paired-end library (the length of paired-end reads was 150 bp for the short insert library, average insert size of 222 +/− 50 bp), which generated 16,857,646 reads, and one long-insert library (average insert size of 7272 +/− 729 bp), which generated 24,172,042 reads totaling 4946 Mbp of Illumina data. All general aspects of library construction and sequencing were performed at the JGI [19]. The initial draft assembly contained 9 contigs in 8 scaffolds. The initial draft data was assembled with Allpaths, version r38445, and the consensus was computationally shredded into 10 kbp overlapping fake reads (shreds). The Illumina draft data was also assembled with Velvet, version 1.1.05 [24], and the consensus sequences were computationally shredded into 1.5 kbp overlapping fake reads. The Illumina draft data was assembled again with Velvet using the shreds from the first Velvet assembly to guide the next assembly. The consensus from the second Velvet assembly was shredded into 1.5 kbp overlapping fake reads. The fake reads from the Allpaths assembly, both Velvet assemblies, and a subset of the Illumina CLIP paired-end reads were finally assembled using parallel phrap, version 4.24 (High Performance Software, LLC). Possible misassemblies were corrected with manual editing in Consed [25–27]. Gap closure was accomplished using repeat resolution software [Wei Gu, unpublished], and sequencing of bridging PCR fragments with Sanger and/or PacBio technologies [Cliff Han, unpublished]. A total of 21 PCR PacBio consensus sequences were completed to close gaps and to raise the quality of the final sequence.

**Table 2:** Project information

MIGS ID	Property	Term
MIGS 31	Finishing quality	High-Quality Draft
MIGS-28	Libraries used	Illumina Standard (short insert paired-end) and Illumina CLIP (long insert paired-end)
MIGS 29	Sequencing platforms	Illumina HiSeq 2000 (CLIP library); Illumina HiSeq 2000 (Standard library); PacBio
MIGS 31.2	Fold coverage	1237×
MIGS 30	Assemblers	Allpaths r38445 and Velvet 1.1.05
MIGS 32	Gene calling method	Prodigal within JGI Prokaryotic Automatic Annotation Pipeline
	Locus Tag	DEINO
	Genbank ID	ATTJ00000000.1
	GenBank Date of Release	07/09/2013
	GOLD ID	Gp0007971
	BIOPROJECT	PRJNA157079
MIGS 13	Source Material Identifier	EXB L-1957
	Project relevance	DNA repair mechanisms, bioremediation

### Genome annotation

The genome sequence was annotated using the JGI Prokaryotic Automatic Annotation Pipeline [28] and further reviewed using the Integrated Microbial Genomes - Expert Review platform [29]. Genes were predicted using Prodigal [30], followed by a round of manual curation using the JGI GenePRIMP pipeline [31]. The genome sequence was analyzed and released publicly through the Integrated Microbial Genomes platform [32]. BLASTClust was used to identify internal clusters with thresholds of 70% covered length and 30% sequence identity [33]. SignalP [34] and TMHMM [35] were used to predict signal peptides and transmembrane helices, respectively.

## Genome properties

The *D. ficus* KS 0460 genome consists of a 4,019,382 bp sequence which represents six genome partitions: 2.84, 0.49, 0.39, 0.20, 0.098 and 0.007 Mbp (Table 3), consistent with PFGE (Fig. 2d); note, the smallest partition (0.007 Mbp) was too small to resolve by PFGE. The final assembly was based on 4946 Mbp of Illumina draft data, which provided an average of 1237× coverage of the genome. The total genomic GC content was 69.7% and was similar across all but the smallest contig, which contained 62.5% GC. The genome contains 3827 predicted protein-coding genes and 67 RNA-coding genes (total 3894).

**Table 3:** Summary of genome: one chromosome and five plasmids

Label	Size (Mbp)	Topology	INSDC identifier	RefSeq ID
Chromosome	2.84	circular	ATTJ01000001	ATTJ01000001
Megaplasmid 1	0.49	circular	ATTJ01000002	ATTJ01000002
Megaplasmid 2	0.39	circular	ATTJ01000003	ATTJ01000003
Megaplasmid 3	0.20	unknown	ATTJ01000004	ATTJ01000004
Plasmid 1	0.098	circular	ATTJ01000005	ATTJ01000005
Plasmid 2	0.007	circular	ATTJ01000006	ATTJ01000006

## Insights from the genome sequence

**Fig. 3:**
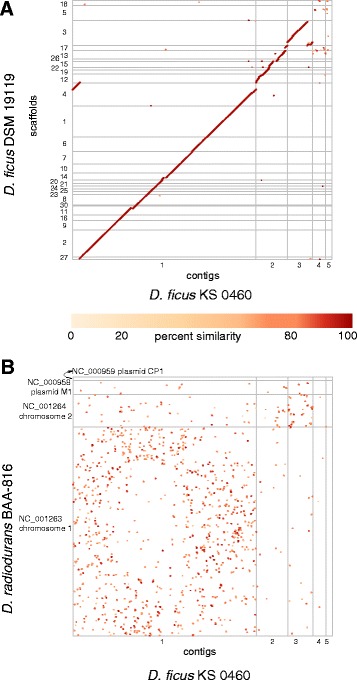
Genomic alignment of *D. ficus* KS 0460 with *D. ficus* DSM 19119 or *D. radiodurans* BAA-816. **a** Strain KS 0460 versus strain DSM 19119. **b** Strain KS 0460 versus strain BAA-816. Six-frame translations of scaffolds were aligned with Mummer 3.23. Homologous regions are plotted as *dots*, colored according to the similarity of the aligned loci. *Diagonal lines of dots* represent syntenic regions. Only contigs longer than 20 kbp are shown. Axes are not drawn to scale

Comparative genomic analysis of strain KS 0460 confirmed the observations made on the basis of the 16S rDNA sequence (Fig. 1) – that the sequenced strain belongs to *D. ficus* and not to *D. grandis*, as originally reported. This is exemplified by the existence of long syntenic regions between the genomes of *D. ficus* strain KS 0460 and the type strain of *D. ficus* DSM 19119 (Fig. 3a), supporting near-identity between the strains; 16S rDNA sequences of these two strains are 99% identical. A close relationship between the strains is also supported by the high (97.8%) genome-wide average nucleotide identity between the two genomes as well as the high (0.84) fraction of orthologous genes (alignment fraction) between them. The suggested cutoff values for average nucleotide identity and alignment fraction between genomes belonging to the same species are 96.5% and 0.60, respectively [36]. The comparison between *D. ficus* KS 0460 and *D. radiodurans* BAA-816 revealed almost no synteny between these genomes (Fig. 3b). Approximately 76% of the predicted proteins contained identifiable Pfam domains, and 72% were assigned to COGs (Tables 4 and 5). Of all *D. ficus* KS 0460 proteins, 3059 and 2717 had homologues in *D. radiodurans* BAA-816 and *D. geothermalis* DSM 11300, respectively. Two regions with coordinates 150,375-159,184 and 2,690,525-2,700,151 on the 2.84 Mbp chromosome [20] were identified as likely prophages of Myoviridae family using PHAST program [37]. The largest number of transposable elements belongs to IS3 family (COG2801). There are 13 copies of this element in the genome. This transposon is absent in the genomes of *D. radiodurans* BAA-816 and *D. geothermalis* DSM 11300.

**Table 4:** Genome statistics

Attribute	Value	% of Total
Genome size (bp)	4,019,382	100.00%
DNA coding (bp)	3,614,725	89.93%
DNA G + C (bp)	2,803,041	69.74%
DNA scaffolds	6	
Total genes	3894	100.00%
Protein coding genes	3827	98.28%
RNA genes	67	1.72%
Pseudo genes	45	1.16%
Genes in internal clusters	982	25.66%
Genes with function prediction	2831	72.7%
Genes assigned to COGs	2747	71.77%
Genes with Pfam domains	2964	76.12%
Genes with signal peptides	458	11.97%
Genes with transmembrane helices	779	20.36%
CRISPR repeats	0	0.00%

**Table 5:** Number of genes associated with general COG functional categories

Code	Value	%age	Description
J	226	6%	Translation, ribosomal structure and biogenesis
A	0	0%	RNA processing and modification
K	166	4%	Transcription
L	97	3%	Replication, recombination and repair
B	0	0%	Chromatin structure and dynamics
D	43	1%	Cell cycle control, Cell division, chromosome partitioning
V	71	2%	Defense mechanisms
T	228	6%	Signal transduction mechanisms
M	146	4%	Cell wall/membrane biogenesis
N	25	1%	Cell motility
U	23	1%	Intracellular trafficking and secretion
O	125	3%	Posttranslational modification, protein turnover, chaperones
C	152	4%	Energy production and conversion
G	179	5%	Carbohydrate transport and metabolism
E	280	7%	Amino acid transport and metabolism
F	90	2%	Nucleotide transport and metabolism
H	149	4%	Coenzyme transport and metabolism
I	116	3%	Lipid transport and metabolism
P	138	4%	Inorganic ion transport and metabolism
Q	58	2%	Secondary metabolites biosynthesis, transport and catabolism
R	217	6%	General function prediction only
S	145	4%	Function unknown
-	1080	28%	Not in COGs

The total is based on the total number of protein coding genes in the genome. Proteins were assigned to the latest updated COG database using the COGnitor program [57]. Other functional categories: defense and mobilome account for 2% and 1%, respectively

### Extended insights

The mapping of *D. ficus* KS 0460 genes to KEGG pathways by KOALA [38] showed that the strain contains the same DNA replication and repair genes as *D. radiodurans*, which were previously shown to be unremarkable [39] (Additional file 2: Table S1). The most striking differences between *D. ficus* KS 0460 and *D. radiodurans* BAA-816 identified by the comparison of the KEGG pathways were in purine degradation and nitrogen metabolism. Specifically, compared to *D. radiodurans*, *D. ficus* lacks guanine deaminase, xanthine dehydrogenase/oxidase, urate oxidase 5-hydroxyisourate hydrolase, 2-oxo-4-hydroxy-4-carboxy-5-ureidoimidazoline decarboxylase, allantoinase, allantoate deiminase, and the entire urease operon (DRA0311-DRA0319 in *D. radiodurans*). In *D. ficus* KS 0460, these metabolic disruptions might contribute to the accumulation of Mn^2+^ antioxidants involved in the protection of proteins from radiation/desiccation-induced ROS [8]. In contrast, *D. ficus* KS 0460 contains eight genes involved in nitrogen metabolism, namely MFS transporter of NNP family, nitrate/nitrite transporter NarK, nitrate reductase/nitrite oxidoreductase alpha subunit, nitrous oxide-forming nitrite reductase, nitrous oxide reductase, nitrite reductase (cytochrome c-5 52), nitronate monooxygenase, hydroxylamine reductase Hcp, and assimilatory nitrate reductase catalytic subunit NapA, that *D. radiodurans* BAA-816 lacks. Other genes present in *D. ficus* KS 0460 but absent in *D. radiodurans* BAA-816 are listed in Additional file 3: Table S2.

Despite the high intracellular Mn concentrations of *Deinococcus* species (Fig. 2f), one of the proteins missing in *D. ficus* KS 0460 is the homologue of the *D. radiodurans* *nramp* Mn-transporter (DR1709), previously identified as critical to extreme IR resistance [40, 41]. On the other hand, *D. ficus* KS 0460 encodes a manganese/zinc/iron ABC transport system (KEGG Module M00319) that is also encoded in the *D. radiodurans* genome. This points to the existence of diverse genetic routes to the complex phenotype of extreme IR resistance even if the physico-chemical defense mechanisms (accumulation of Mn and small metabolites) may be the same [42].

**Fig. 4:**
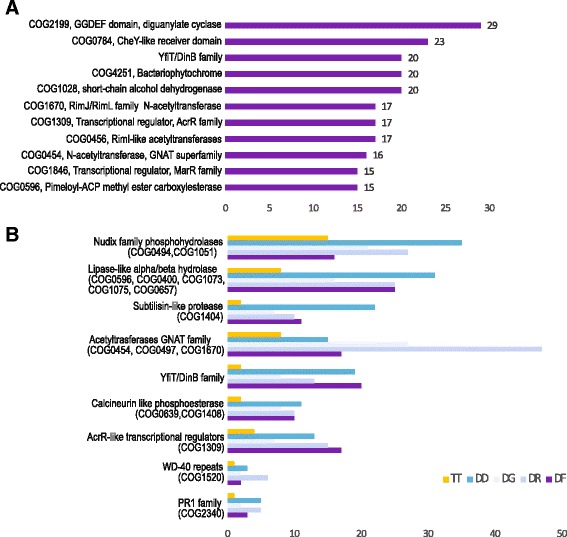
Expanded protein families in *D. ficus* KS 0460. **a** Protein families with 15 or more paralogs in *D. ficus* genome. COG number and family name are indicated on the *left*. **b** Comparison of protein families found to be specifically expanded in *Deinococcus* species. Numbers of proteins correspond to a sum of all COG members indicated in *parenthesis on the left*. Abbreviations: DF, *D. ficus* KS 0460; DR, *D. radiodurans* BAA-816; DG, *D. geothermalis* DSM 11300; DD, *D. deserti* VCD115; TT, *Thermus thermophiles* HB27. Results for DinB/YfiT family were identified using COG2318 and pfam05163

The largest protein families expanded in *D. ficus* KS 0460 include several signal transduction proteins (e.g. CheY-like receiver domains, diguanylate cyclase, bacteriophytochrome-like histidine kinase), several families of acetyltransferases and a stress response protein DinB/YfiT family (Fig. 4a). Many of these families are known to be specifically expanded in previously characterized *Deinococcus* species (Fig. 4b). Thus, *D. ficus* displays the same trend.

In addition to the *nramp* transporter, other genes previously considered to be important to IR resistance are missing in the genome of *D. ficus* KS 0460, namely, the proteins DdrF, DdrJ and DdrK, all of which are also missing in *D. deserti* [3, 40]. DdrO and IrrE proteins found to be key players in regulation of irradiation responses in *D. radiodurans* and *D. deserti* [43, 44] are present in *D. ficus* KS 0460 (DeinoDRAFT_1503 and DeinoDRAFT_1002, respectively). This suggests that the same regulatory pathways are likely active in *D. ficus* KS 0460.

## Conclusions

Twenty years have passed since the extremely IR-resistant bacterium *D. radiodurans* became one of the first free-living organisms to be subjected to whole genome sequencing [45]. Since then, comparative analyses between *D. radiodurans* and other high-quality draft and complete *Deinococcus* genomes have continued, but with few novel findings [10]. *Deinococcus ficus* KS 0460 hereby becomes the eleventh *Deinococcus* reference genome. We confirm by transmission electron microscopy that the very IR-resistant strain KS 0460 grows as single bacillus-shaped cells, whereas deinococci typically grow as diplococci and tetracocci. Our 16S rRNA phylogenetic analysis confirms that strain KS 0460 belongs to the genus *Deinococcus*, its ribosomal RNA being almost identical to the type strain of *D. ficus* DSM 19119. The *D. ficus* KS 0460 genome (4.019 Mbp) is 28% larger than *D. radiodurans* BAA-816 and is divided into six genome partitions compared to four partitions in *D. radiodurans*. Of the 875 genes representing the core genome of ten *Deinococcus* species, only five genes are missing from *D. ficus* KS 0460. In other words, *D. ficus* KS 0460 exemplifies the *Deinococcus* lineage. In particular, *D. ficus* KS 0460 contains the same DNA replication and repair genes, and antioxidant genes (e.g. Mn-dependent superoxide dismutase and catalase) as *D. radiodurans*, which were previously shown to be unremarkable [10]. The most striking genomic differences between *D. ficus* KS 0460 and *D. radiodurans* BAA-816 are metabolic: (i) *D. ficus* lacks nine genes involved in purine degradation present in *D. radiodurans*, possibly contributing to the accumulation of small metabolites known to be involved in the production of Mn^2+^ antioxidants, which specifically protect proteins from IR-induced ROS; and (ii) *D. ficus* contains eight genes in nitrogen metabolism that are absent from *D. radiodurans*, including nitrate and nitrite reductases, suggesting that *D. ficus* has the ability to reduce nitrate, which could facilitate survival in anaerobic/microaerophilic environments. We also show that *D. ficus* KS 0460 accumulates high Mn concentrations and has a significantly higher antioxidant capacity than IR-sensitive bacteria. However, *D. ficus* KS 0460 lacks the homologue of the *D. radiodurans* *nramp* Mn-transporter, previously identified as critical to extreme IR resistance [40, 41], but *D. ficus* KS 0460 encodes at least one alternative manganese transport system. Thus, like previous *Deinococcus* genome comparisons, our *D. ficus* analysis demonstrates the limited ability of genomics to predict complex phenotypes, with the pool of genes consistently present in radioresistant, but absent from radiosensitive species of the phylum shrinking further [3, 10]. With *D. ficus* KS 0460, the number of completed *Deinococcus* genomes is now sufficiently large to determine the core genome and pangenome of these remarkable bacteria. We anticipate that these fresh genomic insights will facilitate approaches applying *Deinococcus* Mn antioxidants in the production of irradiated vaccines [46, 47] and as in vivo radioprotectors [48].

## Additional files

Additional file 1: Figure S1. 16S rRNA phylogenetic tree of the *Deinococcus* genus. The multiple alignment of 16S rRNA sequences was constructed using MUSCLE program [58] with default parameters. The maximum-likelihood phylogenetic tree was reconstructed using the PHYML program [18], with GTR substitution matrix, empirical base frequencies, and gamma-distributed site rates; support values were computed using the aBayes method. *Truepera radiovictrix* was chosen as an outgroup. *D. ficus* KS 0460 is marked in red, *D. ficus* DSM 19119 in green, completely sequenced genomes (according to GenBank) in purple. (PDF 416 kb)

Additional file 2: Table S1. DNA repair genes that are present in* D. ficus* KS 0460 and in *D. radiodurans* BAA-816. (XLSX 13 kb)

Additional file 3: Table S2. Genes that are present in *D. ficus* KS 0460 but absent in *D. radiodurans* BAA-816. (XLSX 44 kb)

## Abbreviations

COG﻿s: Clusters of Orthologous Groups
D_10_: Dose yielding 10% survival
IR: Ionizing radiation
KOALA: KEGG Orthology And Links Annotation
Mn^2+^: Manganous ions
Net AUC: Net area under the fluorescence decay curve
PFGE: Pulsed-field gel electrophoresis
ROS: Reactive oxygen species
USUHS: Uniformed Services University of the Health Sciences
