# Supplementary material for: High-quality genome sequence of the radioresistant bacterium Deinococcus ficus KS 0460
Source: Stand Genomic Sci. 2017 Jul 28;12:46. doi: 10.1186/s40793-017-0258-y (PMC5534035; doi:10.1186/s40793-017-0258-y)
Supplement: Supplementary file 1 — 16S rRNA phylogenetic tree of the Deinococcus genus. The multiple alignment of 16S rRNA sequences was constructed using MUSCLE program [58] with default parameters. The maximum-likelihood phylogenetic tree was reconstructed using the PHYML program [18], with GTR substitution matrix, empirical base frequencies, and gamma-distributed site rates; support values were computed using the aBayes method. Truepera radiovictrix was chosen as an outgroup. D. ficus KS 0460 is marked in red, D. ficus DSM 19119 in green, completely sequenced genomes (according to GenBank) in purple. (PDF 416 kb) [file 40793_2017_258_MOESM1_ESM.pdf]

Figure S1

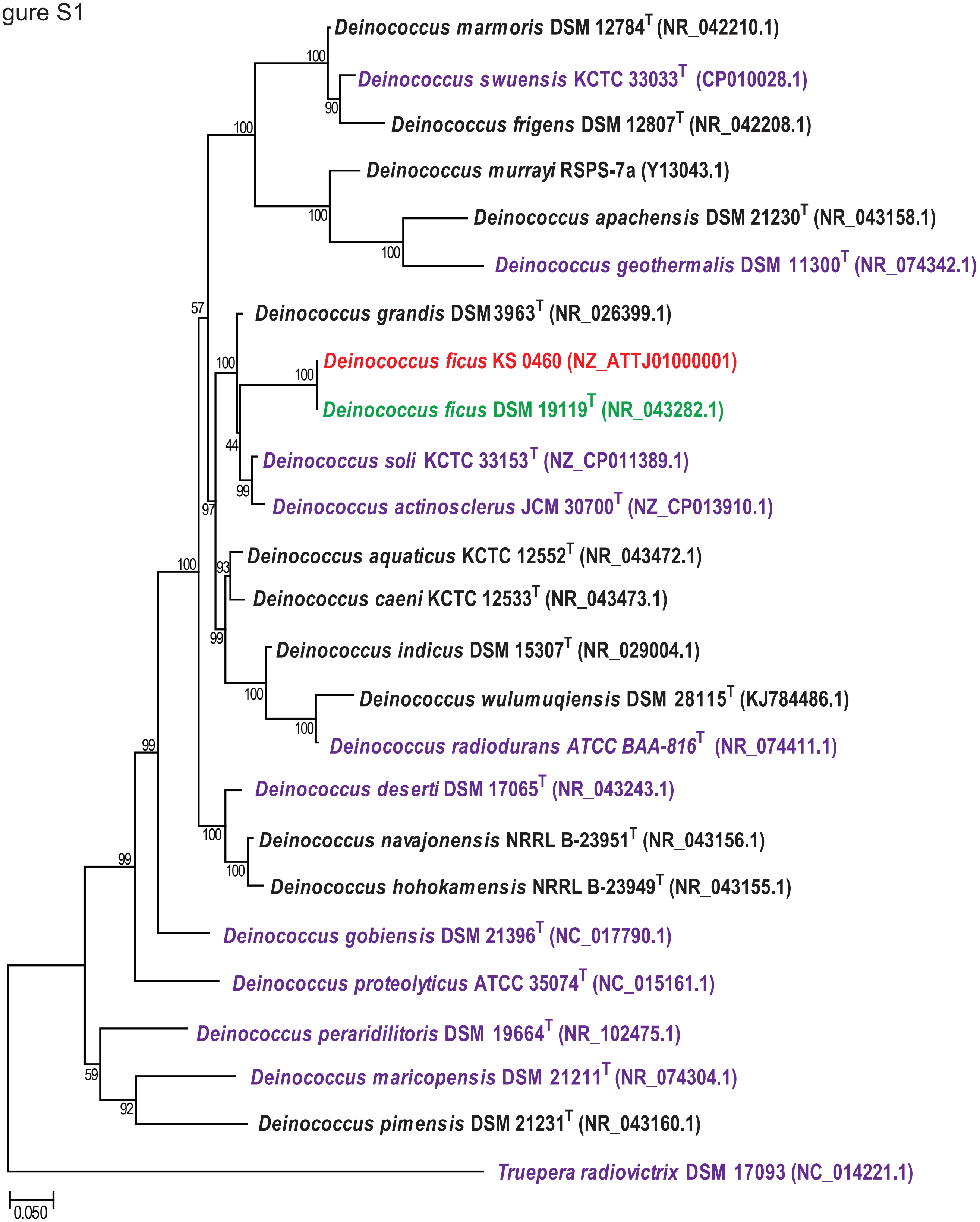

Figure S1. 16S rRNA phylogenetic tree of the *Deinococcus* genus. The multiple alignment of 16S rRNA sequences was constructed using MUSCLE program [58] with default parameters. The maximum-likelihood phylogenetic tree was reconstructed using the PHYML program [18], with GTR substitution matrix, empirical base frequencies, and gamma-distributed site rates; support values were computed using the aBayes method. *Truepera radiovictrix* was chosen as an outgroup. *D. ficus* KS 0460 is marked in red, *D. ficus* DSM 19119 in green, completely sequenced genomes (according to GenBank) in purple.
